# Computation of biological conductance with Liouville quantum master equation

**DOI:** 10.1038/s41598-024-70348-z

**Published:** 2024-08-23

**Authors:** Eszter Papp, Gábor Vattay

**Affiliations:** Department of Physics of Complex Systems, Eötvös Loránd University, Egyetem tér 1-3., Budapest, 1053 Hungary

**Keywords:** Electron transfer, Quantum chemistry, Molecular electronics

## Abstract

Recent experiments have revealed that single proteins can display high conductivity, which stays finite for low temperatures, decays slowly with distance, and exhibits a rich spatial structure featuring highly conducting and strongly insulating domains. Here, we intruduce a new formula by combining the density matrix of the Liouville-Master Equation simulating quantum transport in nanoscale devices, and the phenomenological model of electronic conductance through molecules, that can account for the observed distance- and temperature dependence of conductance in proteins. We demonstrate its efficacy on experimentally highly conductive extracellular cytochrome nanowires, which are good candidates to illustrate our new approach by calculating and visualizing their electronic wiring, given the interest in the arrangement of their conducting and insulating parts. As proteins and protein nanowires exhibit significant potential for diverse applications, including energy production and sensing, our computational technique can accelerate the design of nano-bioelectronic devices.

## Introduction

Protein electron transport measurements exhibit unique properties, making them excellent subjects for in-depth study and exploration^1,2^. When electrodes are attached to protein structures, the measured conductance is surprisingly high, reaching nanoSiemens even over several nanometers of distance^3–5^. It is also noteworthy that the conductance remains stable even when the temperature changes from tens of Kelvins to ambient temperatures^6–8^ and does not show significant decay with increasing protein size between the electrodes^4,5,8^. In bioelectronic measurements, where metallic contacts are chemically bound to molecules, molecular junctions are formed. The Landauer-Büttiker formula is a theoretical tool that accurately describes coherent elastic quantum transport, expressing the conductance in terms of the scattering matrix elements between metallic leads^9^. However, this formula is not applicable at high temperatures, and electron transfer is typically addressed through the semiclassical Marcus theory^10^. It is important to note that both theories are only limiting cases, and electron-vibrational (electron-phonon) interactions should be treated with care in the intermediate regime.

Here, we introduce a new, computationally accessible phenomenological approach that enables us to determine the conductance between atomic orbitals even in the intermediate temperature ranges and can pinpoint areas of high conductivity and insulation in any protein. We build on previous results^11,12^, where a quantum master equation has been used to calculate the electron transfer rate. The novelty is that using the approach developed in Refs.^13,14^ for the electric current in molecules, we derive a new formula connecting the master equation and the conductance. We then use the Liouville master equation introduced by Gebauer and Car^15–17^ for the reduced density matrix of electrons to describe electron transport in nanoscale systems. Its main advantage compared to other quantum master equations, such as the Lindblad and the Redfield equations^18^, is that in the absence of external perturbations, it drives the system toward the correct Fermi-Dirac distribution $F(E,\mu )=1/(1+e^{(E-\mu )/kT})$. This feature makes the Liouville master equation a more appropriate starting point for calculating conductance in molecules. The result is a formula where the conductance is given in terms of the matrix elements of the single electron Hamiltonian, the couplings to the contacts, and the spectral density of the phonon bath.

Following Refs.^11,12^, the reduced density matrix $\rho _{nm}$ of an electron in a molecule can be described with the quantum master equation, which includes the Hamiltonian $H_{nm}$ of the molecule in atomic orbital site basis, the escape rate $\Gamma _m/\hbar$ from the site *m*, the external current $J_m$ at the site *m* and the operator $R_{nm}(\rho )$, which is the phenomenological descriptor of the interaction with the phonon bath. This approach neglects cotunneling and is valid in the linear-response regime. The site-based quantum master equation can be transformed into the energy representation

1 $$\begin{aligned} {\dot{\rho }}^{ij}=-\frac{i}{\hbar }(E_i-E_j)\rho ^{ij}- \frac{1}{2\hbar }\sum _r \left( \Gamma ^{ir}\rho ^{rj}+\rho ^{ir}\Gamma ^{rj} \right) +R^{ij}(\rho )+J^{ij}, \end{aligned}$$

with transformed matrix elements in the energy basis $\rho ^{ij}=\sum _{nm}\Psi _n^i\rho _{nm}\Psi ^j_m$, $\Gamma ^{ij}=\sum _n\Psi _n^i\Gamma _n\Psi _n^j$, $J^{ij}=\sum _n\Psi _n^iJ_n\Psi _n^j$ and $R^{ij}=\sum _{nm}\Psi _n^i\Psi _m^jR_{nm}(\rho )$, where $\Psi _n^i$ and $E_i$ are the eigenfunctions and the egienvalues $E_i\Psi _n^i=\sum _m H_{nm}\Psi _m^i$ of the Hamiltonian. Please, see Supplementary Information S1 for details. We assume that the Hamiltonian is an $N\times N$ real symmetric matrix with real eigenvalues and eigenvectors, where *N* is the number of atomic orbitals. The operator $R(\rho )$ describes the interaction with the environment and ensures the correct equilibrium properties of the electron distribution. In the framework of the single electron picture, the following Liouville master equation has been introduced^15,19^ to describe electron transport in nanoscale systems

2 $$\begin{aligned} R^{ij}(\rho )= \big (\delta _{ij}-\rho ^{ij} \big )\sum _p \frac{1}{2\hbar } \big (\gamma ^{ip}+\gamma ^{jp} \big )\rho ^{pp} -\rho ^{ij}\sum _p \frac{1}{2\hbar } \big (\gamma ^{pi}+\gamma ^{pj} \big ) \big (1-\rho ^{pp} \big ), \end{aligned}$$

where $\gamma ^{ij}$ are the transition rates between the energy levels. This equation can account for the exclusion principle, and in the absence of external currents and escape, its equilibrium solution is the Fermi-Dirac distribution $\rho ^{ij}_{eq}=\delta _{ij}F(E_i,\mu )$, where $\mu$ is the chemical potential of the system. Transition rates between the electron levels are

3 $$\begin{aligned} \gamma ^{ij}=\gamma (\omega _{ij}) \sum _{n} \big |\Psi _n^i \big |^2 \big |\Psi _n^j \big |^2, \end{aligned}$$

where $\omega _{ij}=(E_i-E_j)/\hbar$ is the transition frequency and $\gamma (\omega )$ is the spectral density of the phonon bath, that obeys the Boltzmann-type detailed balance equation $\gamma (\omega )/\gamma (-\omega )=e^{-\hbar \omega /kT}.$ The spectral density of proteins can be modeled effectively by the Ohmic oscillator bath with cutoff^20^ $\gamma (\omega )=\eta \hbar \omega e^{-|\omega |/\omega _c}/(e^{\hbar \omega /kT}-1).$ The concrete form and parameters will not be used in this paper. For reference, the typical cutoff energy is $\hbar \omega _c\approx 0.0185$ eV, and the parameter $\eta =2\pi E_R/\hbar \omega _c\approx 1.46$ where $E_R$ is the reorganization energy^20^. Note that in order to maintain the validity of the Master equation under conditions of weak coupling between the electrons and phonons, the couplings $\gamma ^{ij}$ should be small in comparison to the energies of the electrons and phonons. Conversely, the couplings $\Gamma ^{ij}$ describe the electrons leaking from the molecule to the electrodes and are not subject to the same limitations.

## Conductance

One can couple contacts to two atomic orbital sites called left (*L*) and right (*R*) and calculate the conductance between them. For the derivation, please see the Supplementary Information S2; here, we summarize only the main steps. We switch on a small voltage difference *U* between the contacts with escape rates $\Gamma _L/\hbar$ and $\Gamma _R/\hbar$, and their chemical potential shifts slightly to $\mu _{L/R}=\mu \pm eU/2$. According to the theory developed in Refs.^13,14^, the occupancy of energy $E_i$ in the left and right contacts changes to

4 $$\begin{aligned} \rho _{eq}^i(\mu _{L/R})\approx \rho _{eq}^i(\mu ) \pm D^i(\mu )eU \end{aligned}$$

where $D^i(\mu )=\int dEf(E,\mu )d_i(E)$, and $f(E,\mu )=\partial _\mu F(E,\mu )$ is the derivative of the Fermi-Dirac distribution. The density of states $d_i(E)=\frac{1}{2\pi }\Gamma ^i/((E-E_i)^2+{(\Gamma ^i/2)^2})$ has a Lorentzian broadening due to the finite lifetime caused by the coupling $\Gamma ^i=\Gamma _L |\Psi _L^i|^2 + \Gamma _R |\Psi _R^i|^2$ from the contacts. The bias in the occupation in the left and right contacts generates a net external current $J^{ij}=(eU/2\hbar )(\Gamma _L^{ij}-\Gamma _R^{ij})[D^j(\mu )+D^i(\mu )]$ that should be countered by a slight change in the density matrix $\delta \rho ^{ij}=\rho ^{ij}-\rho ^{ij}_{eq}$ of the molecule to achieve a stationary state. In leading order, the change of the stationary density matrix satisfies the equation $0=\frac{1}{\hbar }\sum _{pq}L^{ijpq}\delta \rho ^{pq}+J^{ij}$, where the operator *L* is the evolution operator of the density matrix, and the Liouville term is linearized around the equilibrium Fermi-Dirac distribution. In the expression of the linearized operator,

5 $$\begin{aligned} L^{ijpq}=-i \big (E_i-E_j \big )\delta _{ip}\delta _{jq}-\frac{1}{2} \big (\Gamma ^{ip}\delta _{jq}+\delta _{ip}\Gamma ^{qj} \big )+\frac{1}{2}\left( {\tilde{\gamma }}^{ip}+{\tilde{\gamma }}^{jp}\right) \delta _{ij}\delta _{pq} -\frac{1}{2}\sum _r \big ({\tilde{\gamma }}^{ri}+{\tilde{\gamma }}^{rj} \big )\delta _{ip}\delta _{jq}, \end{aligned}$$

new transition rates appear, which are related to the old ones by ${\tilde{\gamma }}^{ij}=\gamma ^{ij}(1-F(E_i,\mu ))/(1-F(E_j,\mu )$. Finally, the conductance is given in terms of the inverse of the linearized evolution operator, the escape rates of the contacts, and the molecular orbitals at the contact points

6 $$\begin{aligned} G=-\frac{e^2\Gamma _L\Gamma _R}{\hbar } \sum _{ijpq} \left\{ \Psi _L^i\Psi _L^j \left[ L^{-1} \right] ^{jipq}\Psi ^p_R\Psi _R^q +\Psi _R^i\Psi _R^j \left[ L^{-1} \right] ^{jipq}\Psi ^p_L\Psi _L^q \right\} D^p(\mu ), \end{aligned}$$

which is a new formula and our main theoretical result here. An earlier version of this formula with more restrictive approximations has been introduced in Ref.^21^ and has successfully been applied to understand the temperature dependence of the current flowing through protein monolayer junctions^22^.

## Conductance calculations

Inverting the $N^2\times N^2$ dimensional matrix of the evolution operator for macromolecules like extracellular cytochrome nanowires with $N\sim 10^4$ atomic orbitals is an elusive task. The eigendecomposition of the inverse of the matrix is dominated by the reciprocal of its smallest eigenvalue, and in Ref.^11^, it has been shown that in donor-bridge-acceptor molecular systems, it is a good approximation. The underlying physical assumption is that the relaxation to equilibrium is faster than the escape of the electrons from the system. In Supplementary Information S3, we calculated the conductance in this approximation. The result consists of three terms describing three distinct mechanisms of the total conductance $G=G_{LB}+G_T+G_M$, where

7 $$\begin{aligned} G_{LB}= & {} \frac{2e^2}{h}T,\end{aligned}$$

8 $$\begin{aligned} G_T= & {} \frac{2e^2}{\hbar }\frac{{Z}_L{Z}_R}{{Z}_L+{Z}_R}, \end{aligned}$$

9 $$\begin{aligned} G_M= & {} \frac{e^2}{h}\left[ \frac{{Z}_L}{{Z}_L+{Z}_R}T_{R}+\frac{{Z}_R}{{Z}_L+{Z}_R}T_{L}\right] . \end{aligned}$$

The first term $G_{LB}$ is temperature independent and gives the Landauer-Büttiker formula, where the transmission between the left and right contacts $T=\sum _k \Gamma _L\Gamma _R|\Psi _{L}^k|^2|\Psi _{R}^k|^2/((\mu -E_k)^2+(\Gamma ^k/2)^2)$ is in the Breit-Wigner approximation^9^. It describes coherent elastic processes. This term is suppressed by tunneling in protein structures since the product is exponentially small $|\Psi _{L}^k|^2|\Psi _{R}^k|^2\sim e^{-{l}_{LR}/{l}_T}$, where $l_{LR}$ is the distance of contacts and $l_T\sim 1$ Å is the tunneling length. The second term $G_T$ describes thermal excitation-based conductance, where the terms ${Z}_{L/R}=\sum _k\Gamma _{L/R}|\Psi _{L/R}^k|^2/4kT\cosh ^2((\mu -E_k)/2kT)$ are proportional with the probability for an electron to get from contact *L* or *R* excited to one of the levels of the molecule. The ratio ${Z}_{L/R}/({Z}_L+{Z}_R)$ is the equilibrium probability that an electron from the molecule ends up in contact *L* or *R*. The combination ${Z}_{L}{Z}_{R}/({Z}_L+{Z}_R)$ is the probability that an electron from contact *L* gets via thermal excitation into the molecule and then from the molecule to contact *R*. The third term, $G_M$, is new and describes the mixed process when the electron tunnels from the contact into the molecule described by the transmission $T_{L/R}=\sum _k \Gamma _{L/R}^2|\Psi _{L/R}^k|^4/((\mu -E_k)^2+(\Gamma ^k/2)^2)$ and ends up in the other contact via a thermal process.

**Figure 1:**
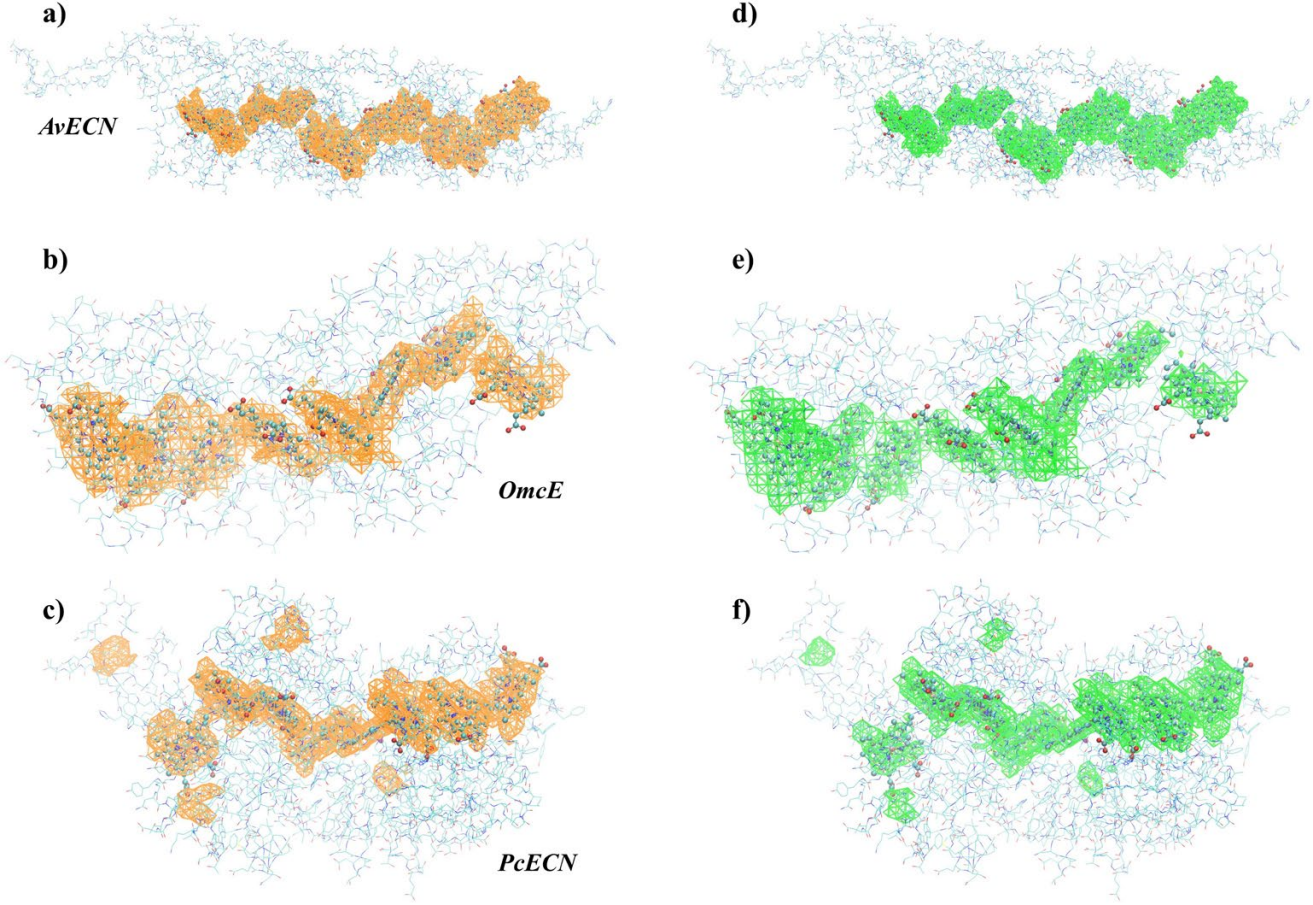
Visualization of the functions ${\mathcal {Z}}(\textbf{r})$ (**a**–**c**) and ${\mathcal {T}}(\textbf{r})$ (**d**–**f**). Highly conductive regions of the molecules are located where both of these functions have high values. On (**a**–**c**), the orange wireframe meshes show the regions where ${\mathcal {Z}}(\textbf{r})$ takes on high values in AvECN (PDB ID: 8E5G), OmcE (PDB ID: 7TFS) and PcECN (PDB ID: 8E5F). (**d**–**f**) shows the same ECNs as on the left side. The green wireframe meshes indicate the regions where ${\mathcal {T}}(\textbf{r})$ takes on high values. Lines represent the amino acids, and the heme molecules are depicted using the ball-stick model. Hydrogen atoms are omitted for simplicity.

The conductance depends on the position of the contacts and the strengths $\Gamma _L$ and $\Gamma _R$. High conductance is achieved potentially when the contacts are on sites with a high *T* or *Z* value. We can visualize the structure by introducing the spatially continuous versions of *T* and *Z*

10 $$\begin{aligned} \mathcal {Z}(\textbf{r})= & {} \sum _k \frac{|\Psi ^k(\textbf{r})|^2}{\cosh ^2((\mu -E_k)/2k_T)}, \end{aligned}$$

11 $$\begin{aligned} \mathcal {T}(\textbf{r})= & {} \sum _k \frac{|\Psi ^k(\textbf{r})|^4}{(\mu -E_k)^2}, \end{aligned}$$

where $\Psi ^k(\textbf{r})$ are the molecular orbitals in space, and the $\Gamma _{L/R}$ dependence has been removed in order to make them independent of the coupling strengths and the position of a second electrode. Highly conducting parts of the molecule are those where $\mathcal {Z}(\textbf{r})$ and $\mathcal {T}(\textbf{r})$ take on high values.

## Extracellular cytochrome nanowires

Certain bacterial species can synthesize conductive protein filaments, also known as bacterial nanowires, to facilitate electron export into extracellular environments for respiration purposes and interspecies electron exchange. One of the extensively studied bacteria is *Geobacter sulfurreducens*, a soil bacterium that produces various types of extracellular cytochrome nanowires (ECNs)^23–26^ that are composed of cytochrome monomers with either 4 (OmcE), 6 (OmcS), or 8 (OmcZ) hemes placed inside the protein, allowing the bacteria to transport electrons over micrometers. OmcE plays a crucial role in extracellular respiration and is also involved in extracellular conductivity^26^. OmcS is essential at the early stages of biofilm growth, direct electron transfer between co-cultures, and Fe(III) oxide reduction^24^. OmcZ nanowires can form a thick conductive biofilm network, possibly because of the branched heme arrangement that leads to one solvent-exposed heme per subunit^27^.

A very recent discovery has brought to light the existence of ECNs in two species of hyperthermophilic archaea, namely Pyrobaculum calidifontis (PcECN) and Archaeoglobus veneficus (AvECN)^28^. These ECNs also serve as mediators for long-range extracellular electron transfer. Although the subunits of ECNs don’t show similarities in their folds, the hemes’ arrangement is common, indicating an evolutionarily optimized structure^28^. It was previously thought that the metal ions were responsible for the long-range electron transport in heme-containing proteins, but recent research indicates that this process is actually dictated by the porphyrin rings^29^. An experimental study combined with density functional theory calculations on a gold-small tetraheme protein-gold junction also reached the same conclusion^30^. While cryo-electron microscopy has enabled the determination of atomic-level structures of these filaments, offering new avenues for theoretical and computational studies, the layout of the conducting and insulating components of ECNs has yet to be determined and visualized. Considering the large size of ECNs, numerical studies that involve entire molecules or oligomers are quite challenging. However, due to their diverse applications^31–35^ and similar structure, ECNs are excellent candidates for investigating the underlying mechanism of electron transport across long biological nanowires. Additionally, measurements display a thousand-fold higher conductivity in OmcZ nanowires compared to OmcS ones^25^, offering a valuable opportunity to assess how well our calculations capture this significant relative difference.

**Figure 2:**
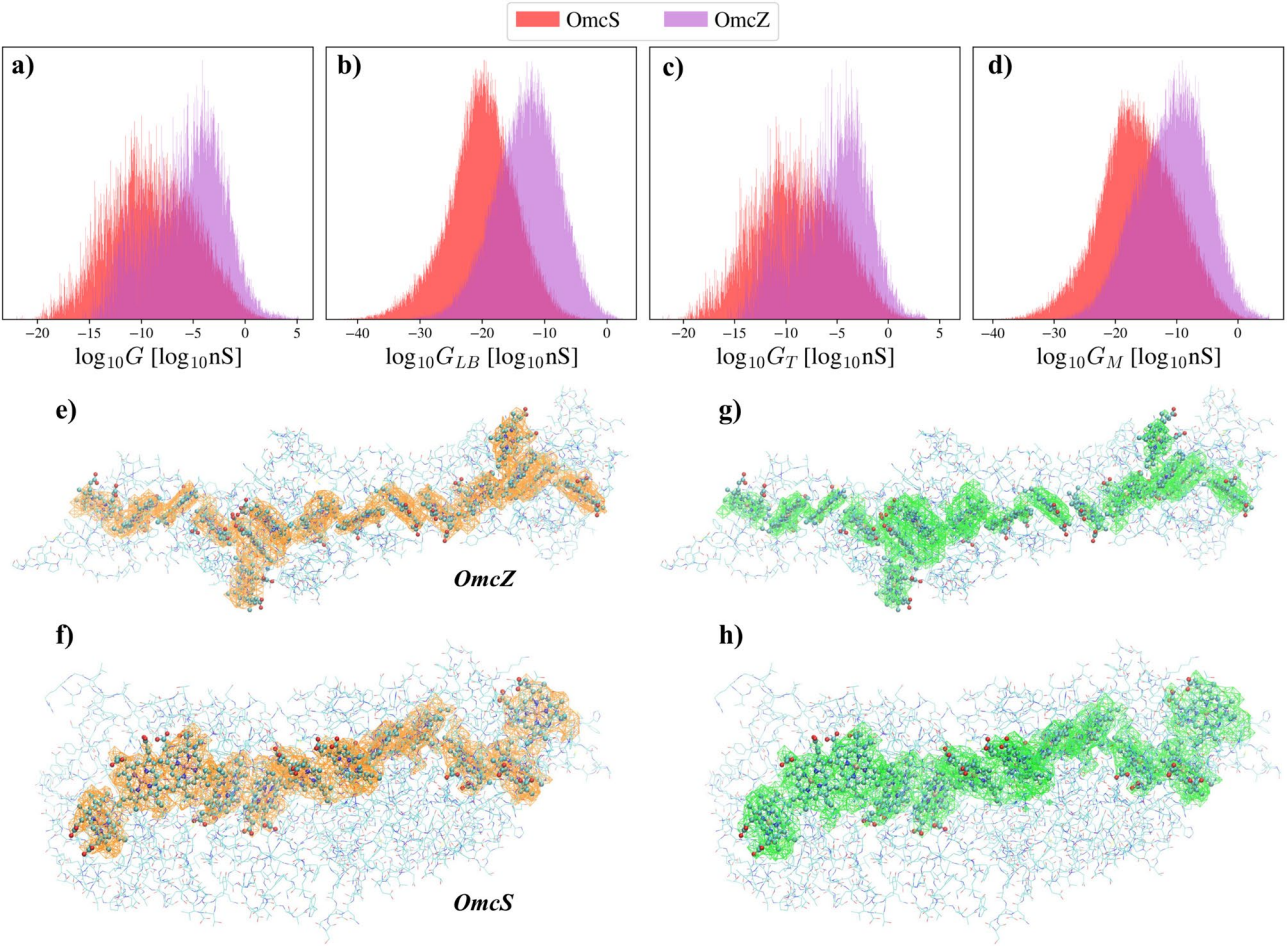
Distributions of the logarithm of the conductance *G* as defined by Eqs. (7–9) for OmcZ and OmcS, calculated with $\Gamma _R = \Gamma _L = 0.1\,eV$ and $T = 300\,K$ (**a**–**d**), and visualization of the functions ${\mathcal {Z}}(\textbf{r})$ (**e**, **f**) and ${\mathcal {T}}(\textbf{r})$ (**g**, **h**) for OmcZ (PDB ID: 7LQ5) and OmcS (PDB ID: 6EF8). The total conductance *G* and all its terms follow a lognormal-like distribution. For both structures, the Landauer-Büttiker term $G_{LB}$ of the conductance (**b**) is the smallest, the dominating term is the thermal excitation-based conductance $G_T$ (**c**), and the last term $G_M$ (**d**) that describes a mixed process, is in between the two. There are several orders of magnitude differences between the means of the total conductance *G* distributions, aligning with experimental observations that show significantly higher values for OmcZ compared to OmcS^25^. On (**e**, **f**), the orange wireframe meshes show the regions where ${\mathcal {Z}}(\textbf{r})$ takes on high values in OmcZ and OmcS. On (**g**, **h**), the green wireframe meshes show the regions where ${\mathcal {T}}(\textbf{r})$ takes on high values in OmcZ and OmcS, respectively. Highly conductive regions are located where both functions have high values. Lines represent the amino acids, and the heme molecules are depicted using the ball-stick model. Hydrogen atoms are omitted for simplicity.

Employing Eqs. (11) and (11), we can visualize the highly conductive regions within the five ECNs introduced here. To represent these regions, we constructed a 3D rectangular grid with 1.5 Å resolution for each ECN structure and computed the values of the functions ${\mathcal {Z}}(\textbf{r})$ and ${\mathcal {T}}(\textbf{r})$ on the grid points at room temperature $kT = 25\,meV$. (See details of the calculations in Methods.) Subsequently, we selected all non-zero values for each function and determined the highest five percent for the function ${\mathcal {Z}}(\textbf{r})$ and the highest twenty percent for the function ${\mathcal {T}}(\textbf{r})$. These percentiles serve as the isovalues for the isosurfaces presented in Figs. 1 and 2e–h.

Consistent resemblances in both ${\mathcal {Z}}(\textbf{r})$ and ${\mathcal {T}}(\textbf{r})$ for all ECNs supports the existence of a common mechanism governing long-range electron transport^28^. ${\mathcal {Z}}(\textbf{r})$ and ${\mathcal {T}}(\textbf{r})$ have similar structures, with the most conductive parts located inside the proteins, spanning across the porphyrin rings, in agreement with previous studies of heme-containing proteins^29,30^. In the case of PcECN (Fig. 1c,f), one can also observe highly conductive regions on the intra-subunit disulfide bonds. The functionality of these additional high-conducting areas is an open question.

Our model also allows us to calculate the conductance between any two atomic orbitals of the ECNs, facilitating a comparison of the conductance values for OmcZ and OmcS. Utilizing Eqs. (7–9), we computed each term ($G_{LB}$, $G_{T}$ and $G_M$) of the total conductance *G* between 100, 000 pairs of randomly selected atomic orbitals for both OmcZ and OmcS at $T=300$ K with parameters $\Gamma _L = \Gamma _R = 0.1$ eV. In addition, we only considered orbitals that are 30–35 Å apart, and do not belong to hydrogen atoms. We show the distributions of the calculated values of *G*, $G_{LB}$, $G_{T}$ and $G_M$ for both OmcS and OmcZ in Fig. 2a–d. Notably, all of the distributions are lognormal-like.

While a detailed quantitative comparison between experimentally measured and computed values is not within the scope of this paper, our calculations reveal several orders of magnitude differences in the conductance of OmcZ and OmcS, consistent with the experimental observation of significantly higher values for OmcZ compared to OmcS. The potential explanation for this difference in the level of structure lies in the distinct heme-heme interactions due to the closer stacking of the hemes in OmcZ^25^. Given the significant reliance of our calculations on individual structures, we arrive at a similar conclusion, that these structural differences are the primary contributors to the observed disparity.

## Distance- and temperature dependence

**Figure 3:**
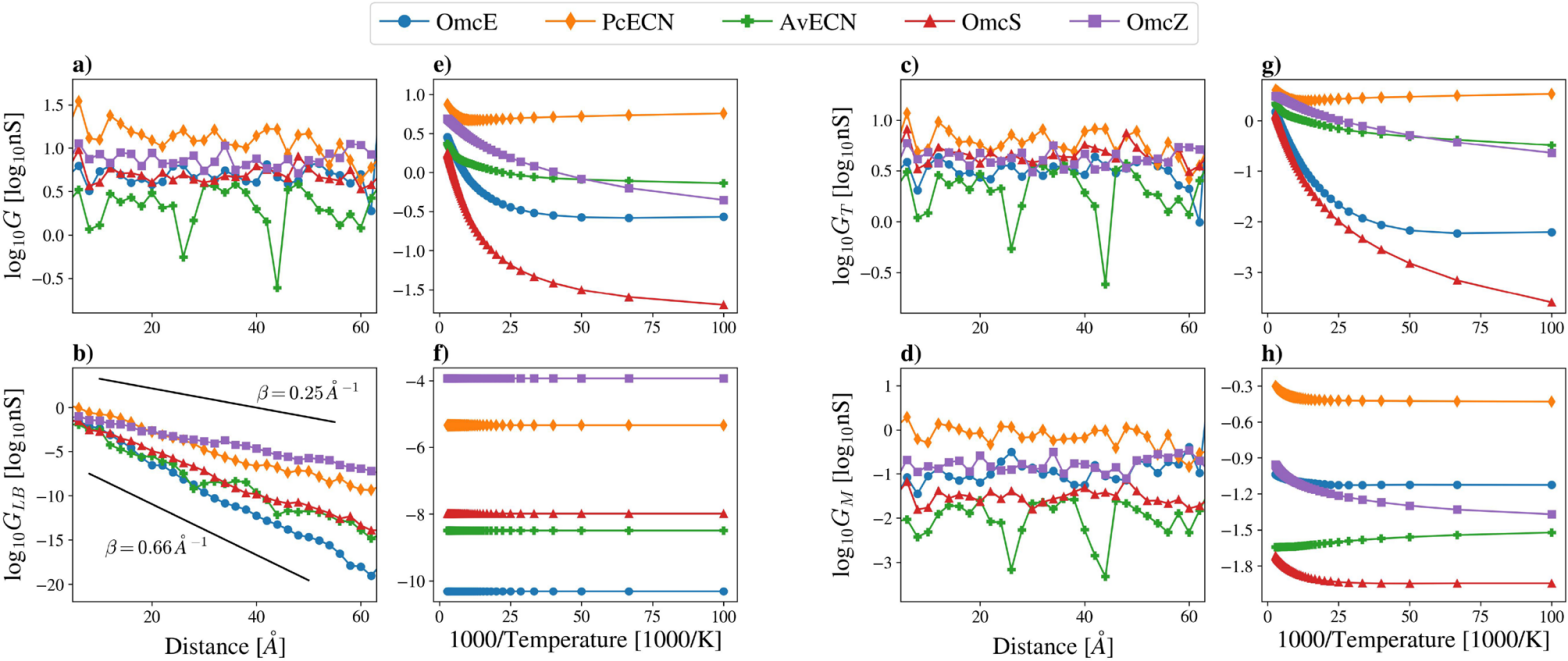
Distance- and temperature dependence of the conductance calculated between atomic orbitals of the ECNs, with coupling strengths $\Gamma _{L}=\Gamma _{R}=0.1\, eV$. (**a**–**d**) shows the distance dependence of the total conductance *G*, the Landauer-Büttiker term $G_{LB}$, the thermal term $G_{T}$ and the mixed term $G_{M}$. On (**e**–**h**), the temperature dependence of the conductance is presented. On (**b**), the two black lines serve as visual guides and follow the form $exp(-\beta d)$, where *d* is the distance between the two atomic orbitals and $1/\beta$ is the decay length of the conductance.

Using Eqs. (7–9), one can study the temperature and distance dependencies of conductance by evaluating it between atomic orbitals positioned at specified distances from each other and at varying temperatures. (See details of the calculations in Methods.) To investigate the distance dependence, the conductance was computed between 400, 000 randomly selected pairs of atomic orbitals belonging to the hemes, which are the most highly conducting parts of the ECNs. The calculation excluded atomic orbitals associated with hydrogen atoms. Then, the mean of the logarithmic conductance values was calculated for each specific distance with a tolerance of $\pm \,  0.1$ Å. The results are presented in Fig. 3a–d. As Fig. 3a shows, the total conductance *G* fluctuates in the $0.1-1\,nS$ range without a clear decreasing trend. A similar pattern is also observed for $G_T$ and $G_M$ fluctuating in various ranges. This reflects the fact that the ratio ${Z}_{L/R}/({Z}_L+{Z}_R)$ associated with the thermal exit probability does not depend on the distance on average. The $G_{LB}$ conductance, which is associated with pure tunneling, decays exponentially with the distance *d* between the two atomic orbitals $G_{LB}\propto exp(-\beta d)$, where the distance decay constant $\beta$ $\approx 0.25-0.66$ Å$^{-1}$. In Ref.^36^, other multiheme cytochrome proteins were investigated, demonstrating a similar result with $\beta = 0.2$ Å$^{-1}$.

Concerning the temperature dependence, a set of 10, 000 pairs of randomly selected atomic orbitals of atoms from the hemes, positioned at distances within the range of 30–35 Å, was considered. Atomic orbitals belonging to hydrogen atoms were excluded from this analysis. Conductance values between these pairs were computed across a temperature range of 10–360 K. The mean of the obtained logarithmic values was calculated for each temperature as shown in Fig. 3e–h. At high temperatures, the transport is clearly thermally activated, and the term $G_T$ dominates the conductance for all ECNs presented here at high temperatures. Interestingly, the dominance shifts to the mixed term $G_M$ in the case of OmcS and OmcE below temperatures $\approx 42$ K and $\approx 67$ K, respectively. As expected, the Landauer-Büttiker term $G_{LB}$ is temperature independent. The mixed tunneling-thermal term $G_M$ falls off with temperature considerably slower than $G_T$ and reaches a finite plateau value at very low temperatures. This is in line with experimental results for several other proteins^6–8,22,37,38^, whereupon cooling, the current through a protein layer exponentially decreases at high temperatures and then reaches a constant value at low temperatures.

In summary, through the integration of the Liouville-Master Equation’s density matrix and a phenomenological model of electronic conductance in molecular systems, we successfully computed and visually represented the conductance attributes of ECNs. Our findings reveal that ECNs resemble insulated cables, characterized by a highly conductive inner core spanning the chain of porphyrin rings within the proteins. This observation aligns with recent experimental findings emphasizing the significance of porphyrin rings within these filaments^29^ and lends support to the proposition that ECN structures have undergone evolutionary optimization for an optimal heme arrangement^28^. We explored the conductance’s and its components’ distance and temperature dependence, finding that the conductance of the studied ECNs falls within the range of 0.1–1 nS with fluctuations but lacks a clear decrease with distance. However, the Landauer-Büttiker term of the total conductance exhibits exponential decay with a decay length of $1/\beta \approx$ 1.5–4 Å, resembling the decay length of $1/\beta = 5$ Å observed in other multiheme cytochromes^36^. Moreover, the simulated temperature-dependence of the conductance in the studied ECNs is consistent with the typical behavior displayed by other proteins^6–8,22,37,38^.

Our newly developed formula possesses the capability to model the conductance of diverse proteins, thereby contributing significantly to the field of nano-bioelectronics. Notably, this formulation incorporates the electron-phonon interaction in the intermediate temperature range, addressing a critical aspect given the non-isolated nature of proteins. This approach enhances the applicability and relevance of our results in advancing the understanding and application of protein electron transport in varied bioelectronic contexts.

## Methods

### Structure preparation

First, we downloaded the cryo-EM structures from the Protein Data Bank (PDB) with PDB IDs 7TFS, 6EF8, 7LQ5, 8E5G and 8E5F for OmcE^26^, OmcS^24^, OmcZ^39^, AvECN^28^ and PcECN^28^, respectively. We created dimers and a trimer from the biological assemblies in the case of AvECN. In Maestro (*Schrödinger Release 2023-4: Maestro, Schrödinger, LLC, New York, NY, 2023.*, https://www.schrodinger.com/platform/products/maestro/), we connected the appropriate cysteines to the hemes and conducted a force-field minimization procedure only on the side chains of those cysteines to optimize the spatial arrangement of the atoms. Then, C-terminal oxygen atoms and missing hydrogen atoms were added to the protein structures.

### Quantum chemistry calculations

After preparing the structures, the Hamiltonian and overlap matrices were calculated. Due to the large size of the proteins, we opted for the Extended Hückel method instead of the more common DFT calculations, which can consider the redox state of each cofactor. Nevertheless, we took the redox states into account by considering the total charge of the protein. The YAeHMOP software (version 3.0.1, https://yaehmop.sourceforge.net) was used for performing the extended Hückel calculations on the molecules, and it requires the positions of the atoms as an input. In the semi-empirical extended Hückel method, the total valence electron wavefunction is described as the product of one-electron wavefunctions:

12 $$\begin{aligned} \Phi _{valence}=\Psi ^1(1)\Psi ^2(2)\Psi ^3(3) \cdots \Psi ^k(n), \end{aligned}$$

where *k* and *n* denote the molecular orbital and the number of the electron, respectively. Each molecular orbital is constructed by Linear Combination of Atomic Orbitals (LCAO):

13 $$\begin{aligned} \Psi ^k=\sum _{r=1}^NW_{r}^{k}\varphi _r.\qquad k=1,2,3, \ldots N. \end{aligned}$$

here $\varphi _r$-s are the valence electrons’ Slater-type atomic orbitals, that form the basis set. The $W_{r}^{k}$ coefficient is the weight of the *r*th atomic orbital in the *k*th molecular orbital. To calculate the coefficients and the spectrum of a molecule, one needs to solve the following generalized eigenvalue problem:

14 $$\begin{aligned} HW=ESW, \end{aligned}$$

where *S* is the overlap matrix, *H* is the Hamiltonian matrix^40^, *E* is the diagonal matrix of molecular orbital energies (energy eigenvalues), and *W* is the matrix of the eigenvectors containing the linear combination coefficients used in the LCAO method. The Hamiltonian and overlap matrices are the outputs of YAeHMOP, and the eigenvalue problem was solved using the SciPy package^41^ in Python. The Highest Occupied Molecular Orbital (HOMO) and the Lowest Unoccupied Molecular Orbital (LUMO) for all structures have been determined automatically based on the total charge of the molecule calculated by the Maestro software (*Schrödinger Release 2023-4: Maestro, Schrödinger, LLC, New York, NY, 2023.*, https://www.schrodinger.com/platform/products/maestro/) at neutral pH. In the case of the OmcS dimer, the HOMO level has been positioned two levels above the automatic value due to the presence of two redundant hydrogen atoms within the structure.

### Visualization

To visualize the coupling strength-independent functions ${\mathcal {Z}}(\textbf{r})$ and ${\mathcal {T}}(\textbf{r})$, it is sufficient to consider molecular orbitals only within $5\,kT$ from HOMO and LUMO. Molecular orbitals were calculated with the presented LCAO method in Python on grids with 1.5 Å resolution. We used the gridData module of the MDAnalysis package^42^ in Python to write input files for the Visual Molecular Dynamics (VMD) molecular visualization program^43,44^.

### Conductance calculation

To calculate the conductance *G*, we performed a Löwdin orthogonalization $\tilde{H} = S^{-1/2}HS^{-1/2}$ first. Subsequently, we computed the conductance between any pair of atomic orbitals in the Löwdin basis using Eqs. (7–9), where $\Psi _{L/R}^k$ is the *k*th molecular orbital at the Löwdin atomic orbital *L*/*R*. To enhance precision, we considered a larger set of molecular orbitals than utilized in the visualization process, specifically $\pm 20$ energy levels above and below the HOMO and LUMO. Molecular orbitals beyond this range have negligible influence on *T*, $T_{L/R}$, and $Z_{L/R}$.

A code workflow chart is provided in the Supplementary Information S4.

### Supplementary Information

Supplementary Information.

## Data Availability

The data generated and analyzed during the current study are available from the corresponding author upon request.

## Code availability

The codes that support the results within this paper are available from the corresponding author upon request.
