# Supplementary material for: Computation of biological conductance with Liouville quantum master equation
Source: Sci Rep. 2024 Aug 23;14:19571. doi: 10.1038/s41598-024-70348-z (PMC11341803; doi:10.1038/s41598-024-70348-z)
Supplement: Supplementary file 1 — Supplementary Information. [file 41598_2024_70348_MOESM1_ESM.pdf]

Supplementary Information:

Computation of Biological Conductance with Liouville Quantum Master Equation

Eszter Papp[1] and Gábor Vattay[1]

Department of Physics of Complex Systems

Eötvös Loránd University, H-1053 Budapest, Egyetem tér 1-3.,

Hungary

(*Electronic mail: gabor.vattay@ttk.elte.hu)

(*Electronic mail: eszter.papp@ttk.elte.hu)

(Dated: 10 July 2024)

S1. MASTER EQUATION FOR ELECTRON TRANSPORT IN MOLECULES

Following Refs.[1,2], the reduced density matrix ρ of an electron in a molecule can be described with the quantum master equation

$$\partial_t \rho = \frac{1}{i\hbar}[H,\rho] + R(\rho), \tag{S1}$$

where H is the Hamiltonian of the molecule and $R(\rho)$ is an operator for the phenomenological description of the interaction with the environmental heat bath. Electrons can also escape from the molecule. This can be taken into account by adding an imaginary part to the site energy $H_{mm} - i\Gamma_m/2$ so that the escape rate from the site m is $\Gamma_m/\hbar$. Electrons can also be added to a site with a rate J_m. The quantum master equation including escape and currents, is

$$\partial_t \rho = \frac{1}{i\hbar}[H,\rho] - \frac{1}{\hbar}\{\Gamma,\rho\} + R(\rho) + J, \tag{S2}$$

where the matrix $\Gamma = \mathrm{diag}\{...,\Gamma_m/2,...\}$ and J is a vector with elements J_m. In site representation, this equation takes the form

$$\dot{\rho}_{nm} = -\frac{i}{\hbar}\sum_k (H_{nk}\rho_{km} - \rho_{nk}H_{km}) - \frac{1}{2\hbar}(\Gamma_n + \Gamma_m)\rho_{nm} + R_{nm}(\rho) + \delta_{nm}J_n, \tag{S3}$$

where $R_{nm}(\rho)$ are the matrix elements of $R(\rho)$ and summations go for all N sites. The site-based quantum master equation can be transformed into energy representation $\hat{\rho}$ with transformed matrix elements $\rho^{ij} = \sum_{nm} \Psi^i_n \rho_{nm} \Psi^j_m$, where Ψ^i_n is the real eigenfunction $E_i \Psi^i_n = \sum_m H_{nm} \Psi^i_m$ of the Hamiltonian. The transformed equation is

$$\dot{\rho}^{ij} = -\frac{i}{\hbar}(E_i - E_j)\rho^{ij} - \frac{1}{2\hbar}\sum_r (\Gamma^{ir}\rho^{rj} + \rho^{ir}\Gamma^{rj}) + R^{ij}(\rho) + J^{ij}, \tag{S4}$$

where $\Gamma^{ij} = \sum_n \Psi^i_n \Gamma_n \Psi^j_n$, $J^{ij} = \sum_n \Psi^i_n J_n \Psi^j_n$ and $R^{ij} = \sum_{nmkl} \Psi^i_n \Psi^j_m R_{nm}(\rho)$. We assumed that the Hamiltonian is an $N \times N$ real symmetric matrix with real eigenvalues and eigenvectors.

The operator $R(\rho)$ should describe the interaction with the environment and ensure the correct equilibrium properties of the electron distribution. In the framework of the single electron picture, the following Liouville master equation[3,4] has been introduced to describe electron transport in nanoscale systems

$$R^{ij}(\rho) = (\delta_{ij} - \rho^{ij})\sum_p \frac{1}{2\hbar}(\gamma^{jp} + \gamma^{jp})\rho^{pp} - \rho^{ij}\sum_p \frac{1}{2\hbar}(\gamma^{pi} + \gamma^{pj})(1 - \rho^{pp}), \tag{S5}$$

which can account for the exclusion principle, and in equilibrium, in the absence of external currents and escape, it leads to the Fermi distribution $\rho^{ij}_{eq} = \delta_{ij}/(1 + e^{(E_i - \mu)/kT})$, where μ is the

chemical potential of the system. For molecules, the electrons are coupled to the phonon bath, which induces transitions between the energy levels with rates

$$\gamma^{ij} = \gamma(\omega_{ij}) \sum_n |\Psi_n^i|^2 |\Psi_n^j|^2, \tag{S6}$$

where $\omega_{ij} = (E_i - E_j)/\hbar$ is the transition frequency and $\gamma(\omega)$ is the spectral density of the bath, and obeys the Boltzmann-type detailed balance equation

$$\gamma^{ij}/\gamma^{ji} = e^{-(E_i - E_j)/kT}. \tag{S7}$$

There are several models used for the approximation of the spectral density for proteins. One of them is the Ohmic oscillator bath with cutoff[5]

$$\gamma(\omega) = \eta \frac{\hbar\omega}{e^{\hbar\omega/kT} - 1} e^{-|\omega|/\omega_c}, \tag{S8}$$

where $\hbar\omega_c \approx 0.0185 eV$ is the typical cutoff frequency for proteins. The parameter $\eta = 2\pi E_R/\hbar\omega_c \approx 1.46$ where E_R is the reorganization energy.

S2. CONDUCTANCE FORMULA

Our starting point is the derivation of Datta et al.'s low-temperature conductance formula for molecules in Refs.6 and 7, which we summarize here briefly. The molecule is coupled to a left and a right electrode. The discrete levels of the molecule E_i are non-resonantly coupled to the left and right electrode sites with indices $n = L$ and $n = R$ with coupling strengths Γ_L and Γ_R, respectively. The presence of contacts broadens the level density, and the energy levels became resonances $E_i - i\Gamma^i/2$, where E_i and Γ^i are given by the real and imaginary parts of the eigenvalues of the Hamiltonian $H_{nm} - i\delta_{nm}\Gamma_n/2$. The local density of states has a Lorentzian form

$$d_i(E) = \frac{1}{2\pi} \frac{\Gamma^i}{(E - E_i)^2 + (\Gamma^i/2)^2}. \tag{S9}$$

For concreteness, here we assume tactically that the contacts can be taken into account perturbatively and in the first order the real parts E_i are unperturbed, while $\Gamma^i = \Gamma_L |\Psi_L^i|^2 + \Gamma_R |\Psi_R^i|^2$.

If the level E_i is in equilibrium with a contact, then its occupation is

$$\rho_{eq}^i(\mu) = 2 \int_{-\infty}^{+\infty} dE d_i(E) F(E, \mu), \tag{S10}$$

where μ is the chemical potential in the contact, $F(E, \mu) = (1 + e^{(E-\mu)/kT})^{-1}$ is the Fermi distribution, and the factor 2 stands for spin degeneracy. The reduced density matrix of the system

in equilibrium $\hat{\rho}_{eq}$ is diagonal $\rho_{eq}^{ij} = \delta_{ij}\rho_{eq}^{i}(\mu)$. If the occupation of the levels differs from the equilibrium with the left contact or with the right contact, then it generates material current out of the left and right contacts

$$J_L = \frac{1}{\hbar}\mathrm{Tr}\{\hat{\Gamma}_L(\hat{\rho}_{eq}(\mu_L) - \hat{\rho})\}, \tag{S11}$$

$$J_R = \frac{1}{\hbar}\mathrm{Tr}\{\hat{\Gamma}_R(\hat{\rho}_{eq}(\mu_R) - \hat{\rho})\}, \tag{S12}$$

where $\mu_{L/R}$ are the chemical potentials of the contacts and the operators $\hat{\Gamma}_{L/R}$ have matrix elements $\Gamma_L^{ij} = \Gamma_L \Psi_L^i \Psi_L^j$ and $\Gamma_R^{ij} = \Gamma_R \Psi_R^i \Psi_R^j$. The two currents are equal and opposite in sign $J_L = -J_R = J$. The electric current is proportional to the material current $I = eJ$. When the electric field is switched on ($U \neq 0$), the chemical potentials at the left and the right contacts differ $\mu_{L/R} = \mu \pm eU/2$, and a net electric current flows. In the linear regime, we can expand the deviation of level occupations from their equilibrium values in the leading order

$$\rho_{eq}^{i}(\mu_{L/R}) \approx 2\int_{-\infty}^{+\infty} dE d_i(E)[F(E,\mu) \pm f(E,\mu)eU/2] = \rho_{eq}^{i}(\mu) \pm D^i(\mu)eU, \tag{S13}$$

where $D^i(\mu) = \int_{-\infty}^{+\infty} f(E,\mu) d_i(E) dE$ and $f(E,\mu) = \partial_\mu F(E,\mu) = 1/4kT\cosh^2((\mu - E)/2kT)$ is the derivative of the Fermi distribution. In molecules, the equilibrium chemical potential is typically between the highest occupied (HOMO) and lowest unoccupied (LUMO) molecular orbitals $E_{HOMO} < \mu < E_{LUMO}$. The derivative of the Fermi distribution is strongly peaked at $E = \mu$ and decays quickly far from the peak. The Lorentzian density of states is strongly peaked at $E = E_i$. Combining these delta function-like behaviors yield

$$D^i(\mu) \approx d_i(\mu) + f(E_i, \mu), \tag{S14}$$

where the first term describes electron tunneling, and the second is the thermal excitation.

The population of the levels in the molecule shift from their equilibrium values. We can introduce the deviation $\delta\hat{\rho} = \hat{\rho} - \hat{\rho}_{eq}$. In leading order, the current can be written as

$$J_L = \frac{1}{\hbar}\mathrm{Tr}(\hat{\Gamma}_L(\hat{D}eU - \delta\hat{\rho})), \tag{S15}$$

$$J_R = -\frac{1}{\hbar}\mathrm{Tr}(\hat{\Gamma}_R(\hat{D}eU + \delta\hat{\rho})), \tag{S16}$$

where the equilibrium value of the density matrix dropped out, and only deviations from the equilibrium remain, and $\hat{D}$ is the operator with matrix elements $D^{ij} = \delta_{ij}D^i(\mu)$. The deviation $\delta\rho^i$ satisfies the stationary version of the master equation (S4)

$$0 = -\frac{i}{\hbar}(E_i - E_j)\delta\rho^{ij} + \delta R^{ij} - \frac{1}{2\hbar}\sum_r(\Gamma^{ir}\delta\rho^{rj} + \delta\rho^{ir}\Gamma^{rj}) + \frac{eU}{2\hbar}(\Gamma_L^{ij} - \Gamma_R^{ij})(D^j + D^i), \tag{S17}$$

where $\delta R^{ij} = R^{ij}(\hat{\rho}_{eq} + \delta\hat{\rho}) - R^{ij}(\hat{\rho}_{eq})$ and we have to keep the terms linear in the deviation only. The linearized expression takes the form

$$\delta R^{ij} = \frac{\delta_{ij}}{2\hbar}\sum_p \left(\frac{1-F_i}{1-F_p}\gamma^{ip} + \frac{1-F_j}{1-F_p}\gamma^{jp}\right)\delta\rho^{pp} - \frac{\delta\rho_{ij}}{2\hbar}\sum_p\left(\frac{1-F_p}{1-F_i}\gamma^{pi} + \frac{1-F_p}{1-F_j}\gamma^{pj}\right), \quad \text{(S18)}$$

where $F_i = (1+e^{(E_i-\mu)/kT})^{-1}$ is the Fermi distribution. We can introduce new transition rates with the definition $\tilde{\gamma}^{ij} = \gamma^{ij}(1-F_i)/(1-F_j)$, and then (S18) takes a simpler form

$$\delta R^{ij} = \frac{\delta_{ij}}{2\hbar}\sum_p \left(\tilde{\gamma}^{ip} + \tilde{\gamma}^{jp}\right)\delta\rho^{pp} - \frac{\delta\rho_{ij}}{2\hbar}\sum_p\left(\tilde{\gamma}^{pi} + \tilde{\gamma}^{pj}\right). \quad \text{(S19)}$$

The new transition rates change the detailed balance (S7) to

$$\tilde{\gamma}^{ij}/\tilde{\gamma}^{ji} = \frac{(1-F_i)^2}{(1-F_j)^2}e^{-(E_i-E_j)/kT} = \frac{\cosh^2((E_j-\mu)/2kT)}{\cosh^2((E_i-\mu)/2kT)}, \quad \text{(S20)}$$

which now reflects the Fermi statistics. If the magnitude of the difference between the chemical potential and the energy is much larger than the thermal energy, then $\cosh^2((E_i-\mu)/2kT) \approx e^{|E_i-\mu|/kT}/4$ and

$$\tilde{\gamma}^{ij}/\tilde{\gamma}^{ji} \approx e^{-(|E_i-\mu|-|E_j-\mu|)/kT}, \quad \text{(S21)}$$

is the Boltzmann-type detailed balance equation reflecting that states below the chemical potential can be regarded as hole states, and $|E_i-\mu|$ is the energy of the hole.

Finally, we can introduce the evolution operator

$$L^{ijpq} = -i(E_i-E_j)\delta_{ip}\delta_{jq} - \frac{1}{2}(\Gamma^{ip}\delta_{jq} + \delta_{ip}\Gamma^{qj}) + \frac{1}{2}\left(\tilde{\gamma}^{ip} + \tilde{\gamma}^{jp}\right)\delta_{ij}\delta_{pq} - \frac{1}{2}\sum_r(\tilde{\gamma}^{ri} + \tilde{\gamma}^{rj})\delta_{ip}\delta_{jq}, \quad \text{(S22)}$$

and write (S18) as

$$0 = \frac{1}{\hbar}\sum_{pq} L^{ijpq}\delta\rho^{pq} + J^{ij}, \quad \text{(S23)}$$

where $\Gamma^{ij} = \Gamma^{ij}_L + \Gamma^{ij}_R$ and $J^{ij} = (eU/2\hbar)(\Gamma^{ij}_L - \Gamma^{ij}_R)(D^j + D^i)$. We can solve (S23) for the density deviation yielding

$$\delta\rho^{ij} = \frac{eU}{2}\sum_{pq}[L^{-1}]^{ijpq}(\Gamma^{pq}_R - \Gamma^{pq}_L)(D^q + D^p). \quad \text{(S24)}$$

We can substitute this back to (S15) and get the material current J_L and electric current $I = eJ_L$ trough the molecule

$$I = \frac{e^2U}{\hbar}\left[\sum_i \Gamma^{ii}_L D^i - \frac{1}{2}\sum_{ijpq}\Gamma^{ij}_L[L^{-1}]^{jipq}(\Gamma^{pq}_R - \Gamma^{pq}_L)(D^q + D^p)\right] \quad \text{(S25)}$$

Probability conservation guarantees that in the absence of external currents the initial probability content of a density matrix is leaving the system through the contacts. Assume that we start with $\rho^{ij}(0) = \delta_{ip}\delta_{jq}$. The material current flowing out through the contacts is given by

$$j(t) = \frac{1}{\hbar}\sum_{ij}\Gamma^{ji}\rho^{ij}(t), \tag{S26}$$

The total probability through the contacts is equal to the initial probability $\int_0^\infty j(t)dt = \mathrm{Tr}(\rho(0)) = \delta_{pq}$. The evolution equation in the absence of external current is

$$\partial_t\rho^{ij} = \frac{1}{\hbar}\sum_{kl}L^{ijkl}\rho^{kl}, \tag{S27}$$

and its solution is $\rho^{ij}(t) = \sum_{kl}\left[\exp(Lt/\hbar)\right]^{ijkl}\rho^{kl}(0)$. Substituting this solution into (S26) and integrating in time yields

$$-\sum_{ij}\Gamma^{ji}\left[L^{-1}\right]^{ijpq} = -\sum_{ij}(\Gamma_L^{ij} + \Gamma_R^{ij})[L^{-1}]^{ijpq} = \delta_{pq}. \tag{S28}$$

Using this relation, we can write the conductance $G = I/U$ in a manifestly symmetric form

$$G = -\frac{e^2\Gamma_L\Gamma_R}{\hbar}\sum_{ijpq}\{\Psi_L^i\Psi_L^j[L^{-1}]^{jipq}\Psi_R^p\Psi_R^q + \Psi_R^i\Psi_R^j[L^{-1}]^{jipq}\Psi_L^p\Psi_L^q\}D^p(\mu), \tag{S29}$$

which is the main result and allows the calculation of the conductance in terms of the resolvent (S23) of the evolution operator.

S3. PERTURBATIVE TREATMENT

Numerical inversion of the $N^2 \times N^2$ matrix of the evolution operator is a daunting task, therefore, we develop a perturbative approximation that significantly reduces the computational effort and allows an analytic treatment if certain conditions are met. We can introduce the left and the right eigenfunctions of the evolution operator

$$\lambda_k V_k^{ij} = \sum_{pq}L^{ijpq}V_k^{pq}, \tag{S30}$$

$$\lambda_k U_k^{ij} = \sum_{pq}L^{pqij}U_k^{pq}. \tag{S31}$$

The inverse of the operator can be expressed in terms of the eigenfunctions and eigenvalues

$$[L^{-1}]^{ijpq} = \sum_{k=0}^{\infty}\frac{1}{\lambda_k}V_k^{ij}U_k^{pq}, \tag{S32}$$

so that terms with nearly zero $\lambda \approx 0$ eigenvalues dominate the inverse. For a closed system $\Gamma^{ij} = 0$ the evolution operator has an equilibrium solution with zero eigenvalue $\lambda_0 = 0$ and

$$0 = \sum_{pq} L^{ijpq} V_0^{pq}, \tag{S33}$$

$$0 = \sum_{pq} L^{pqij} U_0^{pq}, \tag{S34}$$

and the inverse is singular. The real part of the second-largest eigenvalue $\Re(\lambda_1) = -\hbar/\tau_{rlx}$ determines the rate of the relaxation to equilibrium. If the system is open $\Gamma^{ij} \neq 0$, the largest eigenvalue $\lambda_0 = -\hbar/\tau_{esc}$ determines the rate of the escape of electrons leaking out of the system. If we regard the couplings Γ^{ij} as perturbations of the equilibrium, we can calculate the largest eigenvalue in the first order of perturbation theory as the expectation value of the perturbation in equilibrium

$$\lambda_0 = -\frac{1}{2} \sum_{ijpq} U_0^{ij} (\Gamma^{ip} \delta_{jq} + \delta_{ip} \Gamma^{qj}) V_0^{pq}, \tag{S35}$$

where U_0^{ij} and V_0^{pq} are the left and right eigenfunctions of the operator

$$L_0^{ijpq} = -i(E_i - E_j)\delta_{ip}\delta_{jq} + \frac{1}{2}\left(\tilde{\gamma}^{ip} + \tilde{\gamma}^{jp}\right)\delta_{ij}\delta_{pq} - \frac{1}{2}\sum_r (\tilde{\gamma}^{ri} + \tilde{\gamma}^{rj})\delta_{ip}\delta_{jq}. \tag{S36}$$

The left eigenfunction is $U_0^{ij} = \delta_{ij}$ due to probability conservation. The right eigenfunction can be determined from the detailed balance condition $V_0^{ii}\tilde{\gamma}^{ji} = \tilde{\gamma}^{ij} V_0^{jj}$ and normalization. Due to the detailed balance (S20) we get

$$\frac{V_0^{ii}}{V_0^{jj}} = \frac{\cosh^2((\mu - E_j)/2kT)}{\cosh^2((\mu - E_i)/2kT)}, \tag{S37}$$

and the normalized eigenvector is

$$V_0^{ij} = \frac{\delta_{ij}}{\mathscr{N}} \frac{1}{\cosh^2((\mu - E_i)/2kT)}, \tag{S38}$$

where $\mathscr{N} = \sum_k 1/\cosh^2((\mu - E_k)/2kT)$ is the normalization factor. Using these eigenvectors in (S35) we get

$$\lambda_0 = -\frac{1}{\mathscr{N}} \sum_k \frac{\Gamma^{kk}}{\cosh^2((\mu - E_k)/2kT)}. \tag{S39}$$

As long as the relaxation to equilibrium is much faster than the escape time $\tau_{esc} \gg \tau_{rlx}$, we can use the perturbative first eigenvalue and can neglect the rest of the eigenvalues in the expression of the inverse, and we get

$$[L^{-1}]^{ijpq} \approx \frac{1}{\lambda_0} V_0^{ij} U_0^{pq} \approx -\frac{1}{\sum_k \Gamma^{kk}/\cosh^2((\mu - E_k)/2kT)} \frac{\delta_{ij}\delta_{pq}}{\cosh^2((\mu - E_i)/2kT)}. \tag{S40}$$

Substituting this expression into the conductance formula (S29) and using (S14) yields the conductance

$$G = \frac{2e^2}{h}T + \frac{2e^2}{\hbar}\frac{Z_L Z_R}{Z_L + Z_R} + \frac{e^2}{h}\left[\frac{Z_L}{Z_L + Z_R}T_R + \frac{Z_R}{Z_L + Z_R}T_L\right] \tag{S41}$$

where

$$Z_L = \sum_k \frac{\Gamma_L |\Psi_L^k|^2}{4kT\cosh^2((\mu - E_k)/2kT)}, \tag{S42}$$

$$Z_R = \sum_k \frac{\Gamma_R |\Psi_R^k|^2}{4kT\cosh^2((\mu - E_k)/2kT)}, \tag{S43}$$

and

$$T_L = \sum_k \frac{\Gamma_L^2 |\Psi_L^k|^4}{(\mu - E_k)^2 + (\Gamma^k/2)^2}, \tag{S44}$$

$$T_R = \sum_k \frac{\Gamma_R^2 |\Psi_R^k|^4}{(\mu - E_k)^2 + (\Gamma^k/2)^2}, \tag{S45}$$

$$T = \sum_k \frac{\Gamma_L \Gamma_R |\Psi_L^k|^2 |\Psi_R^k|^2}{(\mu - E_k)^2 + (\Gamma^k/2)^2}, \tag{S46}$$

where $\Gamma^k = \Gamma_L|\Psi_L^k|^2 + \Gamma_R|\Psi_R^k|^2$.

S4. CODE WORKFLOW

Our starting point is a 3D structure from the Protein Data Bank (https://www.rcsb.org/), which we process with the Maestro software (https://www.schrodinger.com/platform/products/maestro/). We connected the appropriate cysteines to the hemes and conducted a force-field minimization procedure only on the side chains of those cysteines to optimize the spatial arrangement of the atoms. Then, C-terminal oxygen atoms and missing hydrogen atoms were added to the protein structures. The outputs of this software are the XYZ files, which contain all the atoms and their coordinates in 3D. Then, the input files (YAH files) suitable for the Yaehmop software (https://yaehmop.sourceforge.net/) are created with a few lines of Python code. Then, the quantum chemistry calculations were carried out by Yaehmop, using the Extended Hückel method. The outputs are the Hamiltonian and Overlap matrices of the structures in the non-orthogonal Slater-type atomic orbital basis.

A. Conductance calculation

First, we transform the Hamiltonian into an orthogonal basis with Löwdin transformation using `scipy.linalg.fractional_matrix_power` in Python. Then, with `scipy.linalg.eigh`, we solve the eigenvalue problem and get the spectrum and the eigenvectors (molecular orbitals) of the proteins. These two steps are the most resource-consuming ones as they scale with N^3, where N is the number of atomic orbitals of the protein structure. After that, we choose the atomic orbitals that are connected to the left and right electrodes (L, R). Utilizing Eqs.(7-9), we lastly calculate the conductance between these selected atomic orbitals with our own Python code.

B. Visualization

In the original basis, we solve the generalized eigenvalue problem with the Hamiltonian and Overlap matrices in Python with `scipy.linalg.eigh`. We also made a 3D rectangular grid with 1.5 Å resolution with the griData module of the MDAnalysis Python package (https://www.mdanalysis.org/GridDataFormats/). Using the spectrum and the eigenvectors (molecular orbitals), we are able to calculate the functions $\mathscr{T}(\mathbf{r})$ and $\mathscr{X}(\mathbf{r})$ on the grid. With the griData module, we generate DX files that are suitable outputs for the Visual Molecular Dynamics software (https://www.ks.uiuc.edu/Research/vmd/) to visualize volumetric data and the protein structure it-

self in 3D. The conductance calculation and visualization code workflow are summarized in the following chart.

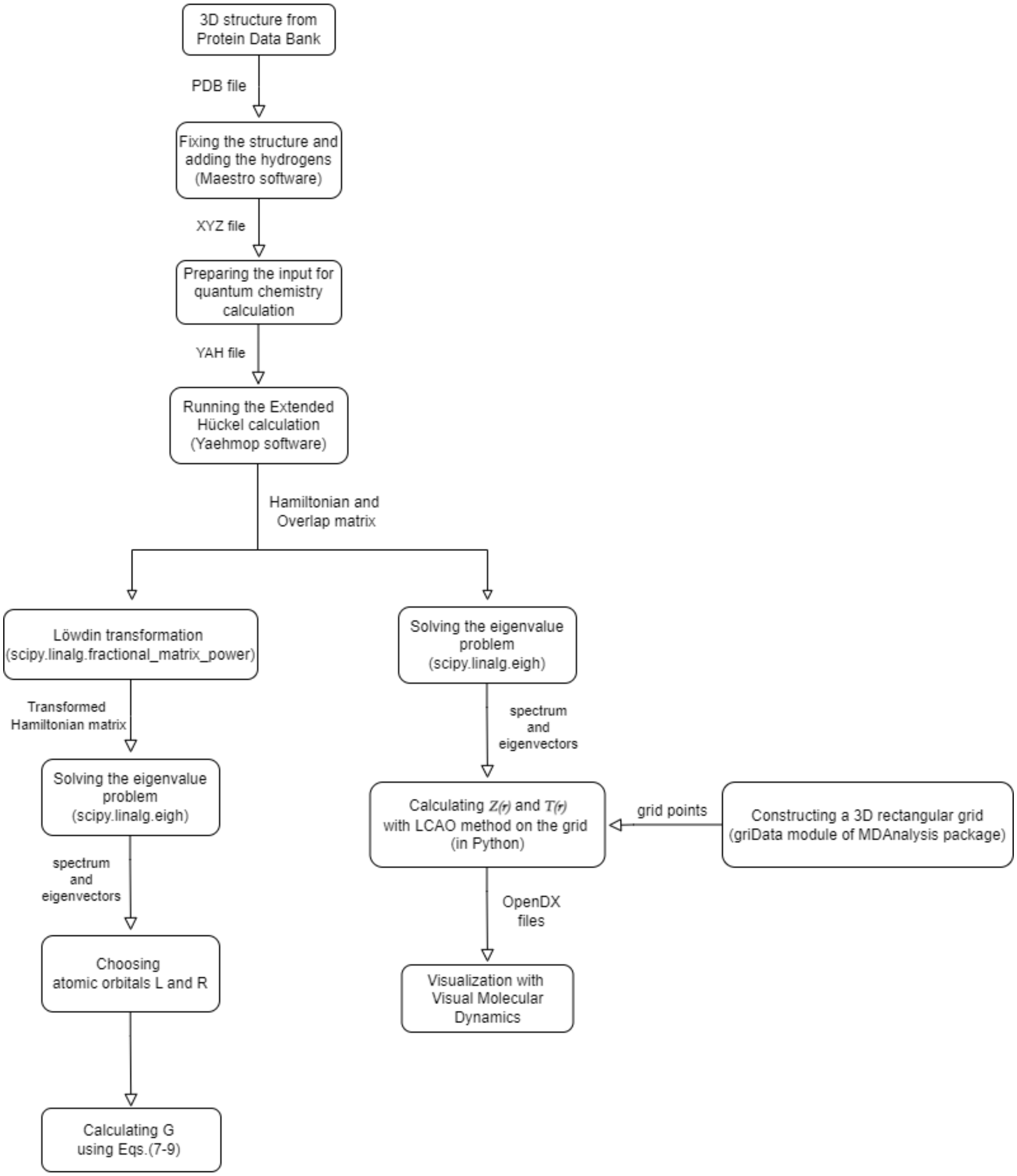

FIG. S1. Code workflow for conductance calculation (left branch) and visualization (right branch).

REFERENCES

[1] W. B. Davis, M. R. Wasielewski, M. A. Ratner, V. Mujica, and A. Nitzan, "Electron transfer rates in bridged molecular systems: a phenomenological approach to relaxation," The Journal of Physical Chemistry A **101**, 6158–6164 (1997).

[2] D. Segal, A. Nitzan, W. B. Davis, M. R. Wasielewski, and M. A. Ratner, "Electron transfer rates in bridged molecular systems 2. a steady-state analysis of coherent tunneling and thermal transitions," The Journal of Physical Chemistry B **104**, 3817–3829 (2000).

[3] R. Gebauer and R. Car, "Kinetic theory of quantum transport at the nanoscale," Physical Review B **70**, 125324 (2004).

[4] R. Gebauer, K. Burke, and R. Car, "Kohn-sham master equation approach to transport through single molecules," in *Time-Dependent Density Functional Theory* (Springer, 2006) pp. 463–477.

[5] M. Mohseni, P. Rebentrost, S. Lloyd, and A. Aspuru-Guzik, "Environment-assisted quantum walks in photosynthetic energy transfer," The Journal of chemical physics **129**, 11B603 (2008).

[6] F. Zahid, M. Paulsson, and S. Datta, "Electrical conduction through molecules," in *Advanced Semiconductor and Organic Nano-Techniques* (Elsevier, 2003) pp. 1–41.

[7] S. Datta, *Electronic transport in mesoscopic systems* (Cambridge university press, 1997).
